# Supplementary material for: The interactome of intact mitochondria by cross-linking mass spectrometry provides evidence for coexisting respiratory supercomplexes
Source: Mol Cell Proteomics. 2017 Dec 8;17(2):216–32. doi: 10.1074/mcp.RA117.000470 (PMC5795388; doi:10.1074/mcp.RA117.000470)
Supplement: Supplemental Data [file supp_RA117.000470_133922_0_supp_23978_fzffwf.zip › spectra_annotation/mito_DR_spectra_annotation/146-1-10-1-29-1.pdf]

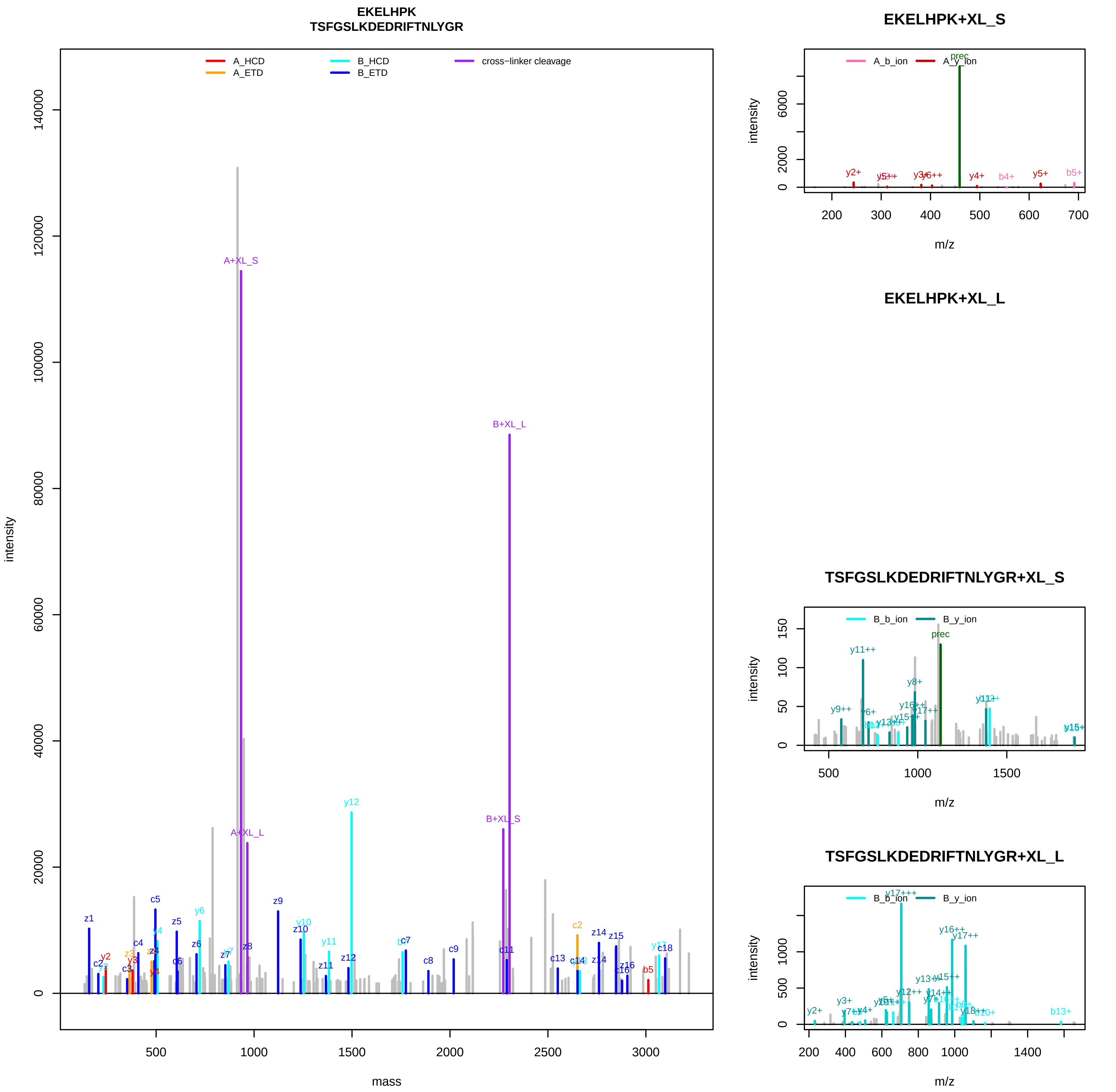
EKELHPK
TSFGSLKDEDRIFTNLYGR
A_HCD
A_ETD
B_HCD
B_ETD
cross−linker cleavage
intensity
mass
0
20000
40000
60000
80000
100000
120000
140000
500
1000
1500
2000
2500
3000
A+XL_S
A+XL_L
B+XL_L
B+XL_S
y12
z1
c2
y2
c3
y3
z3
c4
c5
y4
z4
z5
c6
y6
z6
z7
y7
z8
z9
z10
y10
y11
z11
z12
c7
c8
c9
c11
c13
c14
c2
z14
z15
z16
c16
b5
y17
c18
EKELHPK+XL_S
A_b_ion
A_y_ion
prec
y2+
y5++
y3+
y6++
y4+
b4+
y5+
b5+
m/z
0
2000
6000
200
300
400
500
600
700
EKELHPK+XL_L
TSFGSLKDEDRIFTNLYGR+XL_S
B_b_ion
B_y_ion
prec
y11++
y8+
y9++
y6+
y13++
y15++
y16++
y17++
y11+
y15+
0
50
100
150
500
1000
1500
m/z
TSFGSLKDEDRIFTNLYGR+XL_L
y17+++
y16++
y17++
y13++
y15++
y12++
y14++
y18++
y2+
y3+
y4+
y5+
y7+
b13+
0
500
1000
200
400
600
800
1000
1400
m/z
